# Could continuous subcutaneous glucose sensors (CGMS) be repurposed to diagnose diabetes in equivocal or challenging cases?

**DOI:** 10.1111/dom.16624

**Published:** 2025-07-15

**Authors:** Martin B. Whyte, Adrian H. Heald

**Affiliations:** ^1^ Department of Clinical and Experimental Medicine University of Surrey Guildford UK; ^2^ Department of Diabetes King's College Hospital London UK; ^3^ The School of Medicine and Manchester Academic Health Sciences Centre University of Manchester Manchester UK; ^4^ Department of Diabetes and Endocrinology Salford Royal Hospital Salford UK

**Keywords:** continuous glucose monitoring (CGM), effectiveness, type 1 diabetes, type 2 diabetes

For most of the twentieth century, the measurement of blood glucose after a glucose load was the only generally accepted test to diagnose diabetes. Diagnostic criteria were based on glucose concentrations that best predicted the development of microvascular disease, specifically diabetic retinopathy.^1^ The OGTT has declined in utility because of its low reproducibility and because it is so time‐consuming.^1^ The coefficients of variation can be up to 17% for the 2‐h glucose level.^2^ This inconsistency can result in over 12% being misclassified by the OGTT.^3^

## 1 HbA1c

When first introduced, the inter‐laboratory CV for HbA1c was up to 20%.^4^ This variability led to a standardised approach to HbA1c measurement by the International Federation of Clinical Chemistry and Laboratory Medicine (IFCC). Since then, the inter‐laboratory CV has fallen to ~3.5%.^4^, ^5^ An HbA1c of 6.5% provides almost equal sensitivity and specificity to a fasting or post‐load glucose measurement as a predictor of prevalent retinopathy, and therefore HbA1c was adopted as a diagnostic test by the ADA in 2010.^6^ The excellent assay performance, reasonable cost, and simplicity of measurement (a non‐fasted blood sample) have made HbA1c the preferred measure for diabetes testing at scale. Hence, the means by which diabetes is diagnosed have shifted as technology and measurement accuracy have improved.

However, the utility and practical value of HbA1c as a diagnostic test requires re‐evaluation, considering the known problems of its application, as well as the advent of the continuous glucose monitoring system (CGMS). The sensitivity and specificity of HbA1c can vary with the population studied—for example, being 0.4% lower in the white population than in the black population, with smaller differences observed in Hispanic, Asian, and American Indian people. Factors such as altered red cell turnover, variations in glycation rates, and anaemia can impact HbA1c levels.^1^ There is a very small margin of error that can be tolerated with the HbA1c– even a small positive or negative bias in values can lead to the re‐categorisation of disease state for potentially millions of people. The HbA1c may also fail to detect diabetes in individuals with intermittent or postprandial hyperglycaemia. The 2‐h glucose and HbA1c measure different aspects of glucose metabolism. Decreased insulin secretion, rather than insulin resistance, is a greater factor than HbA1c in the normal or prediabetes range.^7^, ^8^ Consequently, the 2‐h plasma glucose during the OGTT has demonstrated greater sensitivity than HbA1c for diagnosing diabetes in several cohorts.^7^ Hence, a measure of glucose excursion, in equivocal cases, would be of great importance. This may be a niche for CGMS.

## 2 CGMS

CGMS has the potential to overcome these limitations by providing real‐time glucose data and offering a more individualised approach, without the need for a time‐consuming and often unreliable OGTT. It can provide immediate and current insight into glucose trends, including metrics such as mean glucose levels, time in range (TIR), time above range, and glycaemic variability.^9^ These metrics offer a richer understanding of glucose metabolism compared to traditional diagnostic tests. What do CGMS data in people without diabetes look like? Ordinarily, glucose sensors report the target range as 3.9–10.0 mmol/L. However, in people without diabetes, Klonoff et al. (2022) reported that the time spent in a tight glucose range (3.9–7.8 mmol/L) was the most significant distinguishing characteristic of normal glycaemic control, being evident in ~89%–99% of the time.^10^

## 3 USING CGMS FOR EARLY DIAGNOSIS OF TYPE 1 DIABETES

CGMS could initially be used for targeting higher‐risk groups. A number of studies have explored using CGMS to identify the incipient onset of clinical type 1 diabetes (Stage 3) in those with antibody positivity but non‐diabetic glucose ranges. Although, from the wider literature, there is broad correlation of TIR with time in tight range ([TITR], 4–7.8 mmol/L),^11^ in these studies, progression to Stage 3 type 1 diabetes is predicted by a reduced TITR: specifically, CGMS values in those with stage 2 T1D showing at least 18% time spent above 7.8 mmol/L were a key differentiator (80% risk of progression to T1D in 1 year).^12^

## 4 CGMS PROVIDES MEANINGFUL DATA FOR DIAGNOSIS OF TYPE 2 DIABETES

In a study of 100, predominantly Mexican American participants, who were masked to their CGMS data, T2D participants spent more time between 7.8 and 10.0 mmol/L (140 and 180 mg/dL) during the day and overnight, compared with at‐risk and pre‐T2D participants.^13^ This was supported by data from a cohort of over 22 000 people which showed that time in tight range (TTR) discriminates well between those with and without dysglycaemia.^14^ This shows the potential of CGMS to distinguish type 2 diabetes from healthy subjects.

Women with a history of gestational diabetes (GDM) are especially at risk for subsequent T2D. Up to one third of women with GDM will have glucose levels consistent with diabetes or prediabetes on postpartum testing at 6–12 weeks.^15^ In the postpartum period, there is recognized discordance between HbA1c and glucose tolerance test results,^16^ and so repeat OGTT is required.^15^ However, only a quarter of women attend, largely related to women perceiving the test as inconvenient.^15^ CGMS could help overcome the practical difficulties of glycaemic assessment post‐partum, and trials are ongoing.^17^, ^18^

Another approach to using CGMS could be to personalise the interpretation of HbA1c. For example, in individuals with faster red cell turnover or altered glycation rates, CGMS metrics could provide a more accurate estimate of glycaemic control. This is relevant to those receiving haemodialysis, in whom altered red blood cell turnover and/or the use of erythropoiesis‐stimulating agents can lead to falsely low HbA1c values. An individualised approach could mitigate the inaccuracies of HbA1c and improve its reliability as a diagnostic tool. The adjustment of a laboratory measured HbA1c, by incorporating recent CGMS values, can create a personalised HbA1c.^19^

## 5 CHALLENGES

There will be challenges with the adoption of a new paradigm of CGMS data to diagnose diabetes. Current diagnostic criteria for diabetes are based on well‐established thresholds for HbA1c, FPG, and OGTT, but no such thresholds exist for CGMS metrics. The development of standardised, evidence‐based CGMS criteria is essential before CGMS can be used in this capacity. Whilst CGMS technology has improved significantly, it is not as accurate as laboratory‐based plasma glucose measurements at any given point in time. However, thresholds of percentage in a specified range, based upon multiple measures, could mitigate against this. A focus on time in tight glycaemic range could induce anxiety in some users of CGMS.^11^ In others, wearing a sensor might itself lead to behaviour change (the Hawthorne effect), leading to temporary improvements in glucose metrics that are not sustained once observation ends.

The utility and convenience of CGMS compared with measures of plasma glucose for the diagnosis of diabetes need to be balanced against its cost and availability in many countries.

In conclusion, CGMS hold promise to differentiate normal from abnormal glucose handling in people with borderline HbA1c results, bearing in mind that all these parameters exist on a continuum. Its niche could be for the targeting of higher risk groups. The commentary by Cohen and Herman, from the last decade, remains timely: clinicians should not be deterred from using ‘multiple sources of information to assess glycaemia and maintain a high index of suspicion when an indirect measure does not seem correct’.^20^ Let us use these opportunities going forward.

## FUNDING INFORMATION

No funding was received for this article.

## CONFLICT OF INTEREST STATEMENT

The authors declare no conflicts of interest.

## PEER REVIEW

The peer review history for this article is available at https://www.webofscience.com/api/gateway/wos/peer-review/10.1111/dom.16624.

## Data Availability

No new data were used in this commentary.

## References

[1] Genuth S , Alberti KG , Bennett P , et al. Follow‐up report on the diagnosis of diabetes mellitus. Diabetes Care. 2003;26(11):3160‐3167. doi:10.2337/diacare.26.11.3160 14578255

[2] Selvin E , Crainiceanu CM , Brancati FL , Coresh J . Short‐term variability in measures of glycemia and implications for the classification of diabetes. Arch Intern Med. 2007;167(14):1545‐1551. doi:10.1001/archinte.167.14.1545 17646610

[3] Sacks DB . A1C versus glucose testing: a comparison. Diabetes Care. 2011;34(2):518‐523. doi:10.2337/dc10-1546 21270207 PMC3024379

[4] English E , Lenters‐Westra E . HbA1c method performance: the great success story of global standardization. Crit Rev Clin Lab Sci. 2018;55(6):408‐419. doi:10.1080/10408363.2018.1480591 30001673

[5] Goodall I . HbA1c standardisation destination—global IFCC standardisation. How, why, where and when—a tortuous pathway from kit manufacturers, via inter‐laboratory lyophilized and whole blood comparisons to designated national comparison schemes. Clin Biochem Rev. 2005;26(1):5‐19. 16278773 PMC1240025

[6] International Expert Committee . International Expert Committee report on the role of the A1C assay in the diagnosis of diabetes. Diabetes Care. 2009;32(7):1327‐1334. doi:10.2337/dc09-9033 19502545 PMC2699715

[7] American Diabetes Association Professional Practice Committee . Diagnosis and classification of diabetes: standards of care in diabetes‐2024. Diabetes Care. 2024;47(Suppl 1):S20‐S42. doi:10.2337/dc24-S002 38078589 PMC10725812

[8] Bruhn L , Vistisen D , Vaino CTR , Perreault L , Faerch K . Physiological factors contributing to HbA(1c) in the normal and pre‐diabetic range: a cross‐sectional analysis. Endocrine. 2020;68(2):306‐311. doi:10.1007/s12020-020-02234-3 32112239

[9] Lenters‐Westra E , Fokkert M , Kilpatrick ES , et al. Managing discordance between HbA(1c) and glucose management indicator. Diabet Med. 2025;42:e70023. doi:10.1111/dme.70023 40123266 PMC12080991

[10] Klonoff DC , Nguyen KT , Xu NY , Gutierrez A , Espinoza JC , Vidmar AP . Use of continuous glucose monitors by people without diabetes: an idea whose time has come? J Diabetes Sci Technol. 2023;17(6):1686‐1697. doi:10.1177/19322968221110830 35856435 PMC10658694

[11] Beck RW , Raghinaru D , Calhoun P , Bergenstal RM . A comparison of continuous glucose monitoring‐measured time‐in‐range 70–180 mg/dL versus time‐in‐tight‐range 70–140 mg/dL. Diabetes Technol Ther. 2024;26(3):151‐155. doi:10.1089/dia.2023.0380 37870460

[12] Steck AK , Dong F , Taki I , et al. Continuous glucose monitoring predicts progression to diabetes in autoantibody positive children. J Clin Endocrinol Metab. 2019;104(8):3337‐3344. doi:10.1210/jc.2018-02196 30844073 PMC6589073

[13] Barua S , Sabharwal A , Glantz N , et al. Dysglycemia in adults at risk for or living with non‐insulin treated type 2 diabetes: insights from continuous glucose monitoring. EClinMed. 2021;35:100853. doi:10.1016/j.eclinm.2021.100853 PMC8093893 33997745

[14] Dunn TC , Ajjan RA , Bergenstal RM , Xu Y . Is it time to move beyond TIR to TITR? Real‐world data from over 20,000 users of continuous glucose monitoring in patients with type 1 and type 2 diabetes. Diabetes Technol Ther. 2024;26(3):203‐210. doi:10.1089/dia.2023.0565 38444315 PMC10877396

[15] Sweeting A , Wong J , Murphy HR , Ross GP . A clinical update on gestational diabetes mellitus. Endocr Rev. 2022;43(5):763‐793. doi:10.1210/endrev/bnac003 35041752 PMC9512153

[16] Duke A , Yap C , Bradbury R , et al. The discordance between HbA1c and glucose tolerance testing for the postpartum exclusion of diabetes following gestational diabetes. Diabetes Res Clin Pract. 2015;108(1):72‐77. doi:10.1016/j.diabres.2015.01.006 25661662

[17] ClinicalTrials.gov . Postpartum Continuous Glucose Monitoring Study [Internet]. National Library of Medicine (US); 2021. https://clinicaltrials.gov/study/NCT05714761

[18] ClinicalTrials.gov . PREDISPOSE: Predicting Dysglycemia Immediately Postpartum with CGM (NCT04972955) [Internet]. National Library of Medicine (US); 2021. https://clinicaltrials.gov/study/NCT04972955

[19] Heald AH , Stedman M , Paisley A , et al. Consistency of the personalized glycated haemoglobin (pHbA1c) methodology over time in people with type 1 diabetes (T1D) using continuous glucose monitoring. Diabet Med. 2025;42(4):e15520. doi:10.1111/dme.15520 39901499 PMC11929557

[20] Cohen RM , Herman WH . Are glycated serum proteins ready for prime time? Lancet Diabetes Endocrinol. 2014;2(4):264‐265. doi:10.1016/S2213-8587(14)70003-8 24703035

